# An Improved FFR Design with a Ventilation Fan: CFD Simulation and Validation

**DOI:** 10.1371/journal.pone.0159848

**Published:** 2016-07-25

**Authors:** Xiaotie Zhang, Hui Li, Shengnan Shen, Yu Rao, Feng Chen

**Affiliations:** School of Power and Mechanical Engineering, Wuhan University, Wuhan, China; Worcester Polytechnic Institute, UNITED STATES

## Abstract

This article presents an improved Filtering Facepiece Respirator (FFR) designed to increase the comfort of wearers during low-moderate work. The improved FFR aims to lower the deadspace temperature and CO_2_ level by an active ventilation fan. The reversing modeling is used to build the 3D geometric model of this FFR; the Computational Fluid Dynamics (CFD) simulation is then introduced to investigate the flow field. Based on the simulation result, the ventilation fan of the improved FFR can fit the flow field well when placed in the proper blowing orientation; streamlines from this fan show a cup-shape distribution and are perfectly matched to the shape of the FFR and human face when the fan blowing inward. In the deadspace of the improved FFR, the CO_2_ volume fraction is controlled by the optimized flow field. In addition, an experimental prototype of the improved FFR has been tested to validate the simulation. A wireless temperature sensor is used to detect the temperature variation inside the prototype FFR, deadspace temperature is lowered by 2 K compared to the normal FFR without a fan. An infrared camera (IRC) method is used to elucidate the temperature distribution on the prototype FFR's outside surface and the wearer's face, surface temperature is lowered notably. Both inside and outside temperature results from the simulation are in agreement with experimental results. Therefore, adding an inward-blowing fan on the outer surface of an N95 FFR is a feasible approach to reducing the deadspace CO_2_ concentration and improve temperature comfort.

## Introduction

Filtering Facepiece Respirators (FFR) are commonly used to protect users from inhaling contaminant particles. The N95 FFR model, filters out at least 95% of ≥0.3μm particles during testing at a continuous flow rate of 85 L/min, when tested in a laboratory. [1, 2] It is commonly utilized in the industry and health care environment. During the N95 FFR usage, the filter facepiece covers the human face to resist the ingress of particles and an internal cavity called the deadspace is generated. Owning to the resistance of filter fibers, which inhibits the exhaled air from dissipating out; the deadspace air is reported to have a higher temperature and an elevated CO_2_ level. [1–8]

In order to dissipate the heat and CO_2_ from the deadspace of N95 FFRs, a kind of one-way valve called an exhalation valve (EV) is added. This allows for a uni-directional exit of the exhaled air through N95 FFRs while preventing outside air from entering. [9] However, the EV is activated by the positive pressure during exhalations, and the ventilation provided by the EV depends on the respiratory rate. Therefore, it is instead of surprising that the EV is reported to be non-functional at low-moderate work rates owning to the lack of the adequate breathing volume. [1] To improve this, an active ventilation device is proposed to replace or work in cooperation with EVs. [10] The essence of this idea is to replace the breathing pressure-driven valve with a battery-driven fan so that the deadspace ventilation can be ensured at any respiratory rate. Research on this issue is limited; a published CFD study used a model with a simplified axial fan placed across the filter facepiece, and results show that the deadspace CO_2_ level is lowered. [10] However, placing the fan across the filter facepiece destroys the initial structure of an N95 FFR and may introduce new problems, such as the decrease of filter efficiency and the increase of cost.

To efficiently dissipate the exhaled air from the FFR deadspace, this work proposes an improved FFR design with a ventilation fan. The ventilation fan is attached to the outer surface of the filter facepiece without changing its structure. A geometric model including the headform, N95 FFR and fan is rebuilt by the CT scanning and reverse modeling in our previous work. [11] The CFD simulation is used to investigate the flow field, air temperature and CO_2_ concentration in the FFR deadspace. Effects of the ventilation fan on flow characteristics in the FFR deadspace are also investigated. Finally, a wireless temperature sensor and an infrared camera (IRC) are used to measure the temperature inside and outside the FFR, and to validate the CFD simulation approach.

## Materials and Methods

### A) Improved FFR design

**Fig 1:**
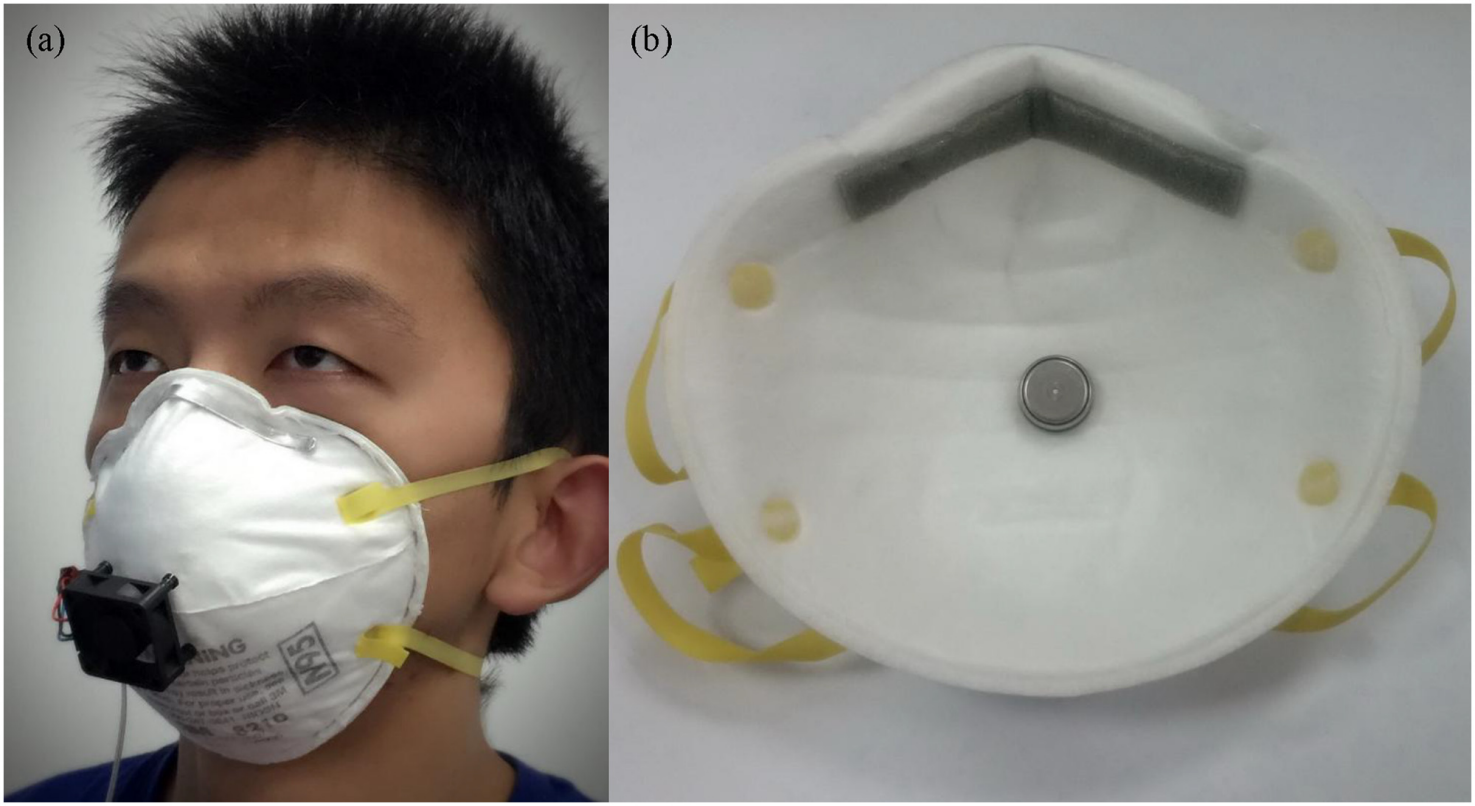
An improved FFR design. (a) the experimental prototype of improved FFR worn by subject (b) sensor position for temperature measurement.

The study was approved by the Human Subjects Review Board of Wuhan University, and the subject provided oral and written informed consent. The individual in this manuscript has given written informed consent (as outlined in PLOS consent form) to publish these case details. Fig 1(a) shows the experimental prototype of improved FFR which combines a normal FFR with the ventilation fan. The ventilation fan is an axial fan placed on the outer surface of a 3M 8210 FFR (3M Corp., St. Paul, MN, USA). It is located in the middle of the filter facepiece, and close to the nostrils and mouth. The outlet of this fan is 1 cm away from the FFR surface. The prototype FFR is normally worn by the same subject in our previous work so that the CFD simulation and the experiment are based on the same model. [11] In addition, the weight of the fan is 10.5 grams, which is too light to affect the shape and position of the worn FFR. Table 1 shows the specific technical details of the ventilation fan.

**Table 1:** Official technical parameters of the ventilation fan: Delta 3010 series. (http://www.delta-china.com.cn/)

MODEL	Rated voltage (V)	Rated current (A)	Rated input power (Watt)	Speed (rpm)	Max air flow (m^3^/min)	Noise (dB-A)
3010 (AFB0305LA)	5	0.07	0.35	6,000	0.067	17.5
3010 (AFB0312LA)	12	0.05	0.60	6,000	0.067	17.5
3010 (AFB0305MA)	5	0.11	0.55	7,500	0.085	20.0
3010 (AFB0312MA)	12	0.07	0.84	7,500	0.085	20.0
3010 (AFB0305HA)	5	0.16	0.80	9,000	0.100	25.0
3010 (AFB0312HA)	12	0.09	1.08	9,000	0.100	25.0

Fig 1(b) shows the wireless temperature sensor used in this study. In order to validate results of the CFD simulation, the FFR deadspace temperature is measured by an I-Button semiconductor temperature sensor (I-Button, Dallas, TX, USA). Its wireless capability and small dimensions (16×6 mm) allow it to be easily embedded into the FFR. In addition, its temperature resolution (up to 0.0625 K) is high enough for this study. The selected temperature sensor is glued to the middle of the inner surface of the FFR, ensuring that it cannot contact the skin of the subject thus avoiding the potential interference caused by the skin temperature. Moreover, a FLIR A655sc (FLIR Systems, Wilsonville, OR, USA) infrared camera is used to detect the surface temperature distribution on the FFR and the subject's face. The study was approved by the Human Subjects Review Board of our University, and the subject provided oral and written informed consent.

### B) CFD simulation method

**Fig 2:**
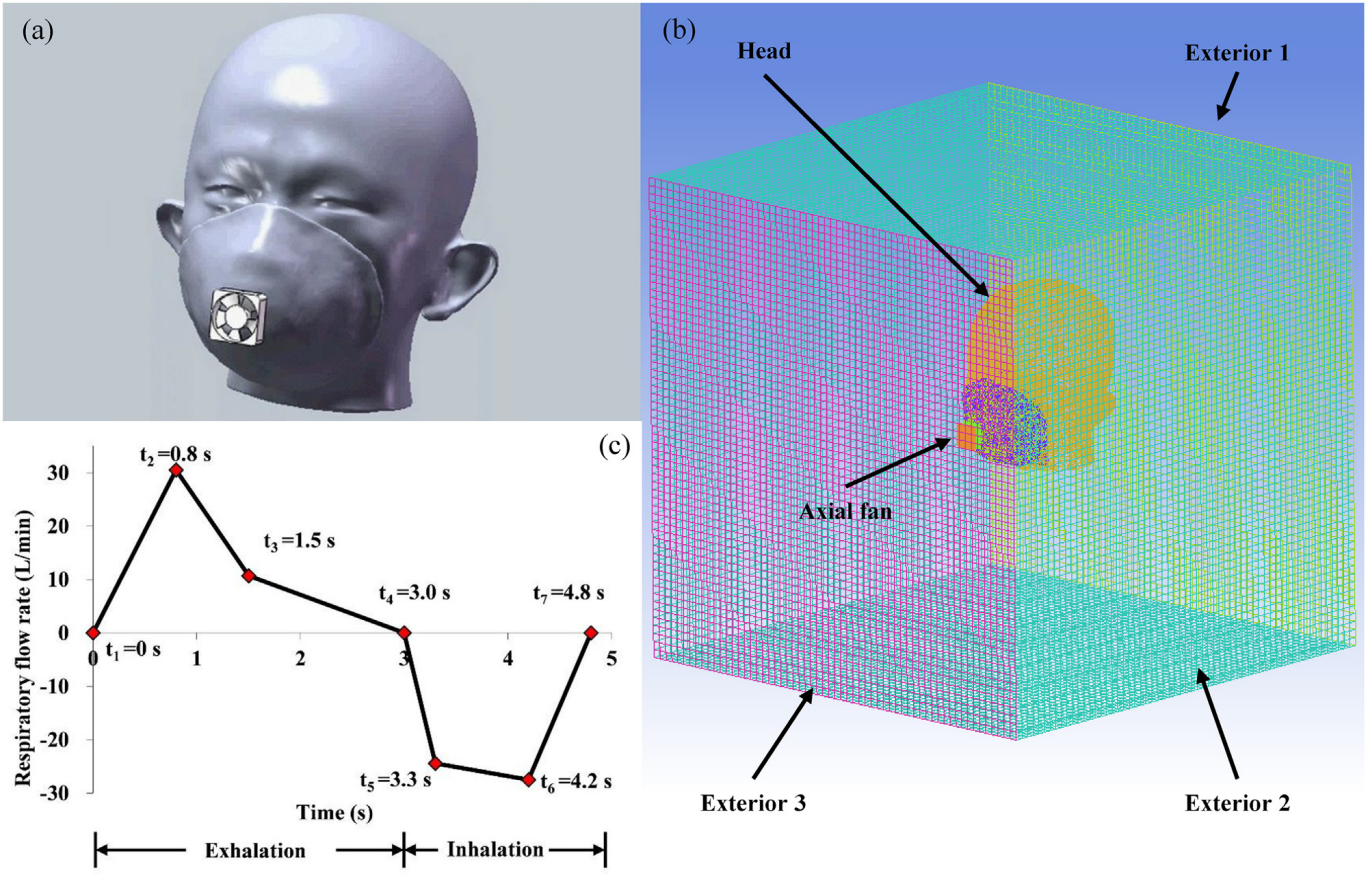
Simulation model: (a) 3D geometric model (b) finite element mesh for CFD simulation (c) the respiratory flow rate.

In order to investigate the flow field using the CFD simulation, an integral geometric model that contains all components including the headform, FFR and axial fan needs to be built preliminarily. Geometric models of the headform and FFR were rebuilt by the CT scanning and reverse modeling in our previous work. [11] Therefore, the modeling, setup and validation of the ventilation fan are new challenges to this study. A geometric model of the axial fan is built according to the dimensions and parameters provided by its manufacturer (Delta Electronics, Taipei, Taiwan, China). Fig 2(a) shows the 3D integral geometric model based on the same subject as in Fig 1(a). Fig 2(b) shows the view of mesh zones in the CFD simulation. The moving mesh model is used to simulate the rotation of the axial fan because it is suitable for the transient simulation. [12] The dimension of the simulation domain is 600×600×600 mm, which is 10 times larger than the headform model to guarantee the simulation accuracy.

**Fig 3:**
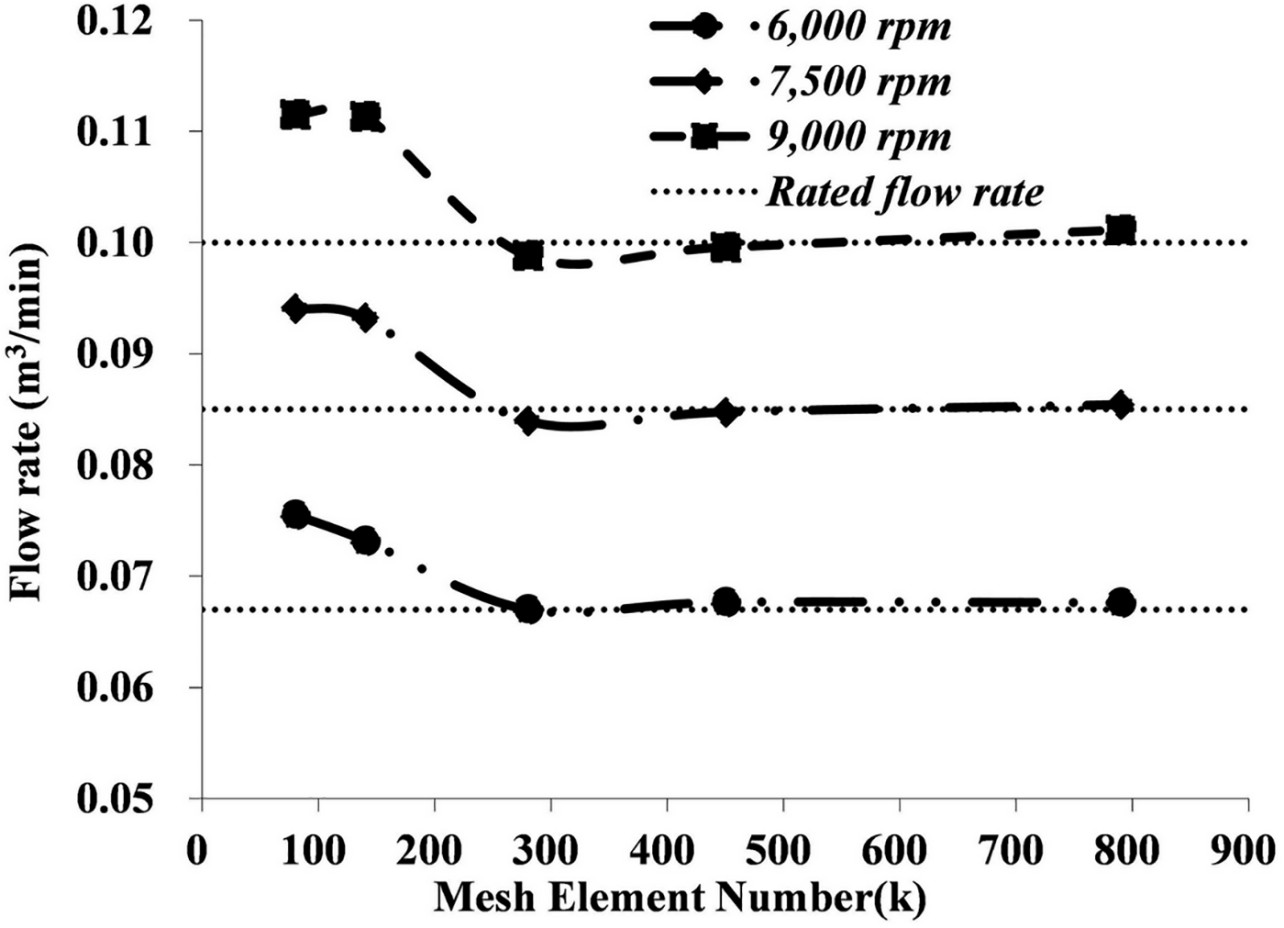
Grid Independency and speed sensibility test of the ventilation fan.

The CFD simulation around the human body has been conducted previously, [13–15] but few models have contained a rotating fan. To ensure the new added fan properly fits our previous CFD model of the respiratory system, [11] five different mesh sizes are tested at three typical rotating speeds to test the grid independency and speed sensibility of the axial fan. Flow rates on surfaces of the fan intake and outtake are monitored and are shown in Fig 3. Results show that 300 thousand discrete elements are sufficient to achieve a stable result regarding the flow rate for all three rotating speeds. In addition, compared to the flow rate data provided by the manufacturer (Table 1), results at the rotation speed of 7,500 rpm show the best consistency with the described flow rate, but results at the other two rotating speeds diverge slightly from that. Therefore, the rotation speed of 7,500 rpm will be used in the subsequent study. The flow field of the N95 FFR with the ventilation fan is simulated using a commercial CFD software package (FLUENT 12.0, ANSYS, Canonsburg, PA, USA). This continuous transient simulation process can be classified thus:

1. For the tidal air flow from the respiratory and constant air flow from the ventilation fan, no high pressure source exists which can lead to the considerable air compression, and the real breathing procedure is time-dependent. Consequently, an unsteady incompressible model was chosen. The RNG (ReNormalization Group) k-e turbulence model was used because it has been proven to be an accurate predictor both in indoor air simulations and respiratory airway simulation. [16–19] In addition, it has also been used in the CFD simulation of the axial fan’s behavior. [12]
2. The heat exchange between the environment air, dead space air, FFR material and different facial layers of the muscle, fat and blood are complex. Simulations of the heat exchange have been implemented previously to detect leakages while wearing an FFR. [16] Research also showed that N95 FFR use resulted in non-significant, minimal increases in the core temperature and uncovered facial skin (cheek) temperature. [4] This work therefore assumes that the main source for the temperature rise is the high-temperature exhaled airflow, not the face or other sources.
3. When modeling the air species transport through the FFR, it is assumed that there is no water phase change (water vapor became liquid water or vice versa) and no chemical reaction. Initially, the ambient air occupies the whole domain at the standard atmospheric pressure. At a temperature of 296.6 K, the standard ambient air composition is calculated to be 0.03% CO_2_, 20.71% O_2_, 1.23% H_2_O and 78.03% N_2_, and the exhaled air composition is calculated to be 4.88% CO_2_, 15.61% O_2_, 2.44% H_2_O and 77.07% N_2_. [20]
4. The filter medium of the FFR is assumed to be a porous medium to simulate its resistance to the air flow, as used in our previous work. [11] In the laminar flow through the porous media, the pressure drop is proportional to the velocity, and the momentum equation is calculated by the Darcy’s law. [21] Table 2 lists the necessary details of the CFD simulation.

**Table 2:** CFD model and boundary conditions.

Basic models	RNG *k-e* turbulence model, species transport model, heat conduction model
Nostril	Velocity inlet: Velocity profile as Fig 2(c), direction normal to the boundary, temperature 307.6 K, turbulent intensity 1%, and hydraulic diameter 10 mm. initial CO_2_ concentration of 4.88%
Exterior 1,2	Pressure outlet: Gauge pressure 0 Pa, backflow temperature 296.6 K, turbulent intensity 1%, length scale 600 mm. initial CO_2_ concentration 0.03%,
Exterior 3	Wall: Slip, temperature 296.6 K.
Head	Wall: No slip; shell conduction, thickness 14–16 mm, inner side temperature 309.6 K.
Axial fan zone	Moving mesh zone, rotating speed: 7,500 rpm
FFR zone	Porous zone, FFR porosity 0.88, viscous resistance coefficient 1.12 × 10^10^,
Initial condition	Velocity is zero; temperature is 296.6 K; turbulent kinetic energy is 10−5 m^2^ / s^2^; turbulent dissipation rate 10−6 m^2^ / s^3^, 0.03% CO_2_, 20.71% O_2_, 1.23% H_2_O and 78.03% N_2_

## Results and Discussion

### A) Flow characteristics of the ventilation fan

**Fig 4:**
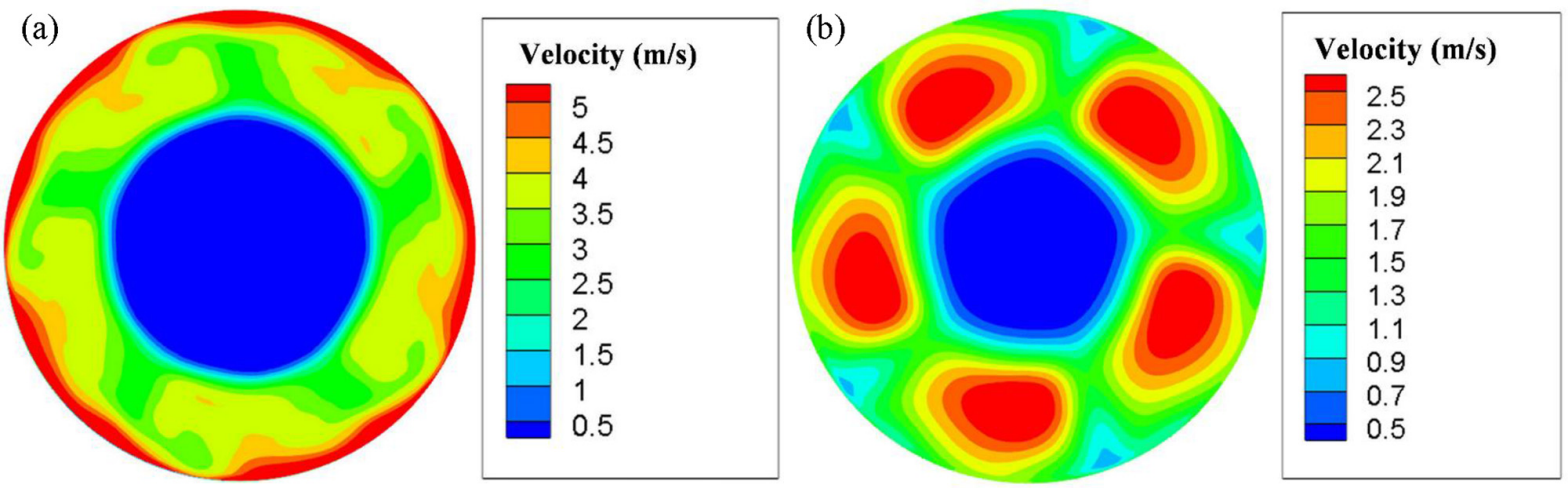
Air velocity contours of an axial fan. (a) Outtake side (b) Intake side.

Fig 4 shows the air velocity distributions on two surfaces representing the intake and outtake of the fan. It can be observed that velocity distributions of the two sides are very different.

On the outtake side as shown in Fig 4(a), large velocities appear at the boundary of the fan frame, where airflows are fiercely compressed. The greatest velocity exceeds 5 m/s. Velocities around fan blades are relatively smaller, at about 3 m/s. The central zone has the lowest velocity, at about 0.5 m/s. On the intake side as shown in Fig 4(b), high velocities are distributed along with the five fan blades, and the greatest velocity is above 2.5 m/s.

### B) Effects of fan orientation

**Fig 5:**
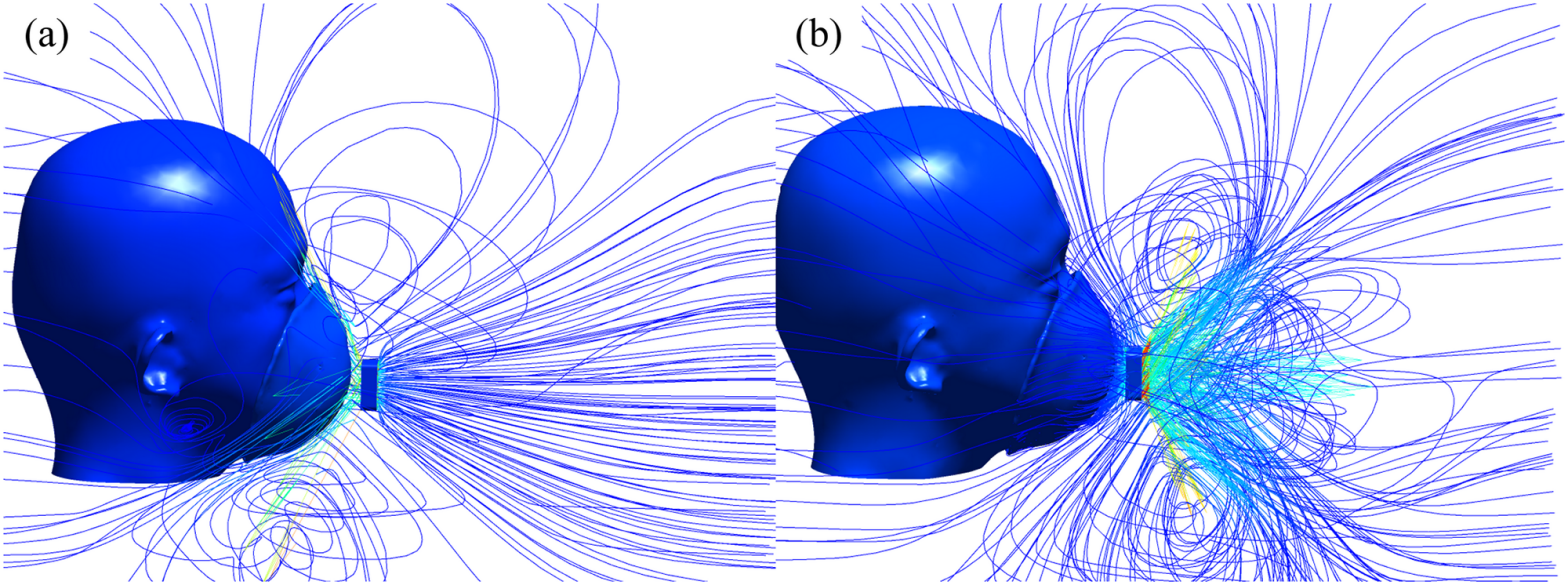
Streamline distribution around the object's head when the FFR with a ventilation fan. (a) Outward-blowing fan (b) Inward-blowing fan.

The orientation of the fan outlet is investigated to ensure its optimum ventilation efficiency. Fig 5 shows the streamline distribution of two opposite orientations, outward-blowing and inward-blowing.

When the fan blows towards the outside of the human face in Fig 5(a), the intake side of the fan (left side) is close to the FFR and airflows are extracted from a relative narrow space into the axial fan. The intake efficiency could therefore be lowered.

In addition, on the outtake side of the fan (right side), the streamline distribution is cup-shaped, which should fit the FFR and human face well. But it is not helpful in the design of the outward-blowing fan in Fig 5(a), since the airflow blows away. However, in Fig 5(b), when the orientation is changed, cup-shaped high speed airflows out from the fan can suitably cover and envelop the FFR and human face. In addition, intake flows are smoothly and evenly distributed in Fig 5(b). So the sufficient airflow is sucked in, and the potentially lowered intake efficiency is avoided.

In order to further confirm the orientation of the FFR fan and its ventilation efficiency, three different cases are simulated, representing cases with and without a ventilation fan, respectively. The average temperature and volume fraction of CO_2_ are monitored and compared.

**Fig 6:**
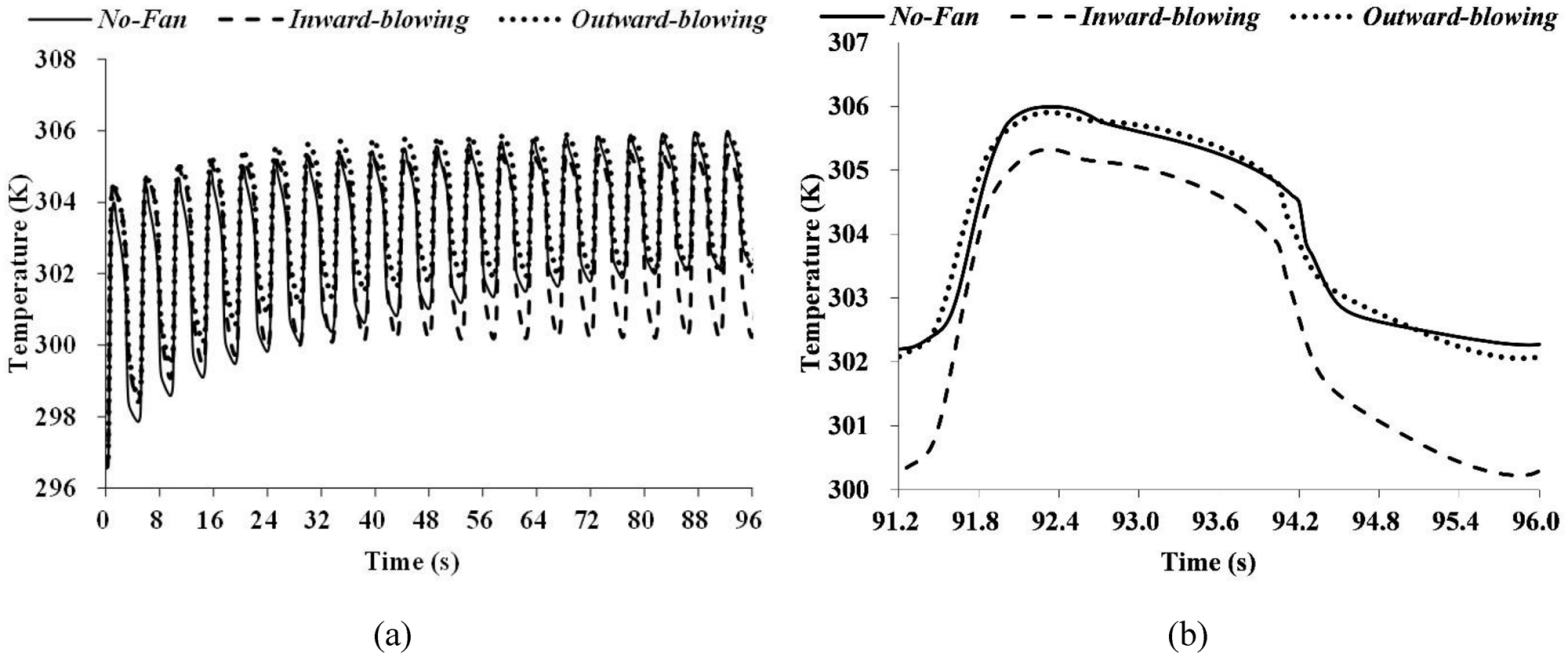
FFR deadspace temperature when FFRs with/without a ventilation fan.

**Fig 7:**
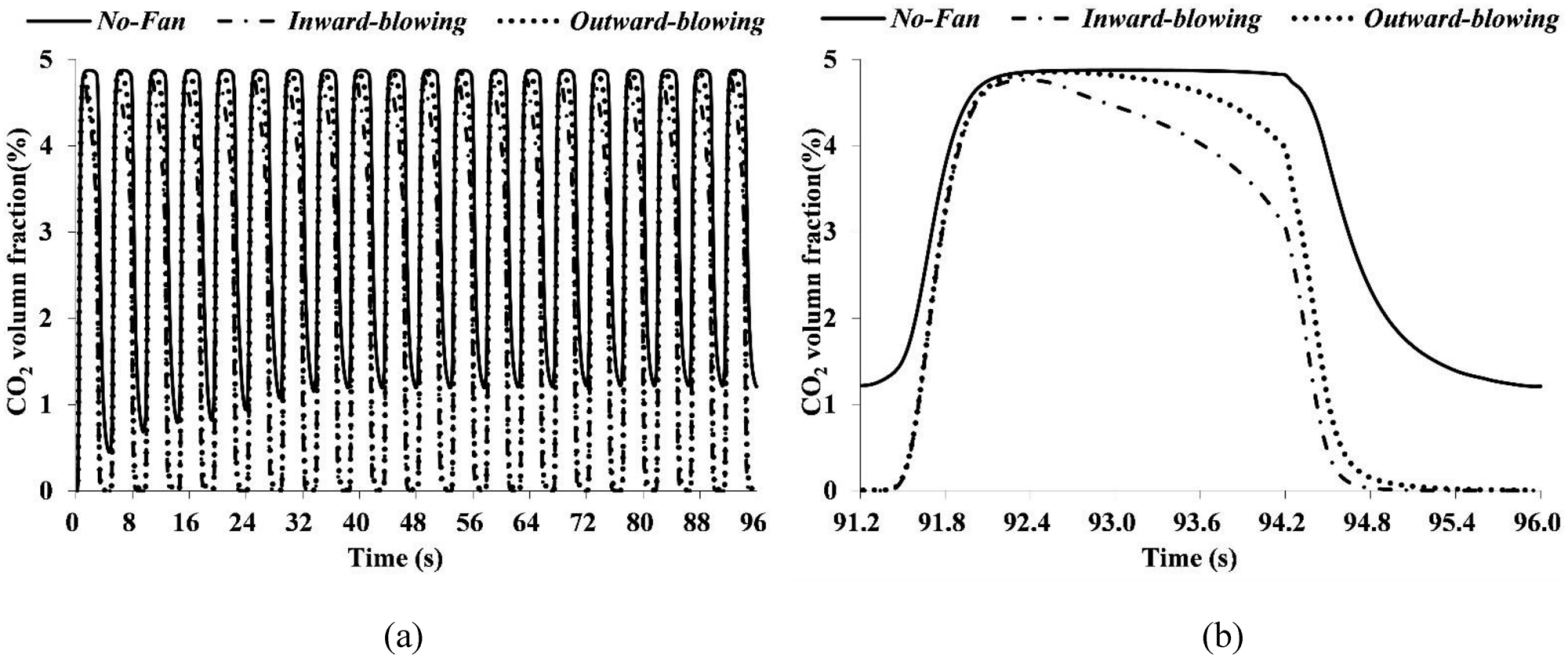
CO2 volume fraction in the FFR deadspace when FFRs used with/without a ventilation fan.

Fig 6(a) shows temperature results after 20 respiration cycles. The inward-blowing fan can notably lower the average temperature inside the FFR deadspace. Fig 6(b) shows the temperature result in a single respiration cycle. Two curves almost overlap, representing that the outward-blowing fan shows no obvious improvement on the heat dissipation. Fig 7 shows the CO_2_ volume fraction in the FFR deadspace. It shows that during first 20 respiration cycles, the axial fan with both orientations can accelerate the CO_2_ dissipation. However, the inward-blowing fan still has a better ventilation efficiency as shown in Fig 7(b).

### C) Experiment on temperature of headform and FFR

**Fig 8:**
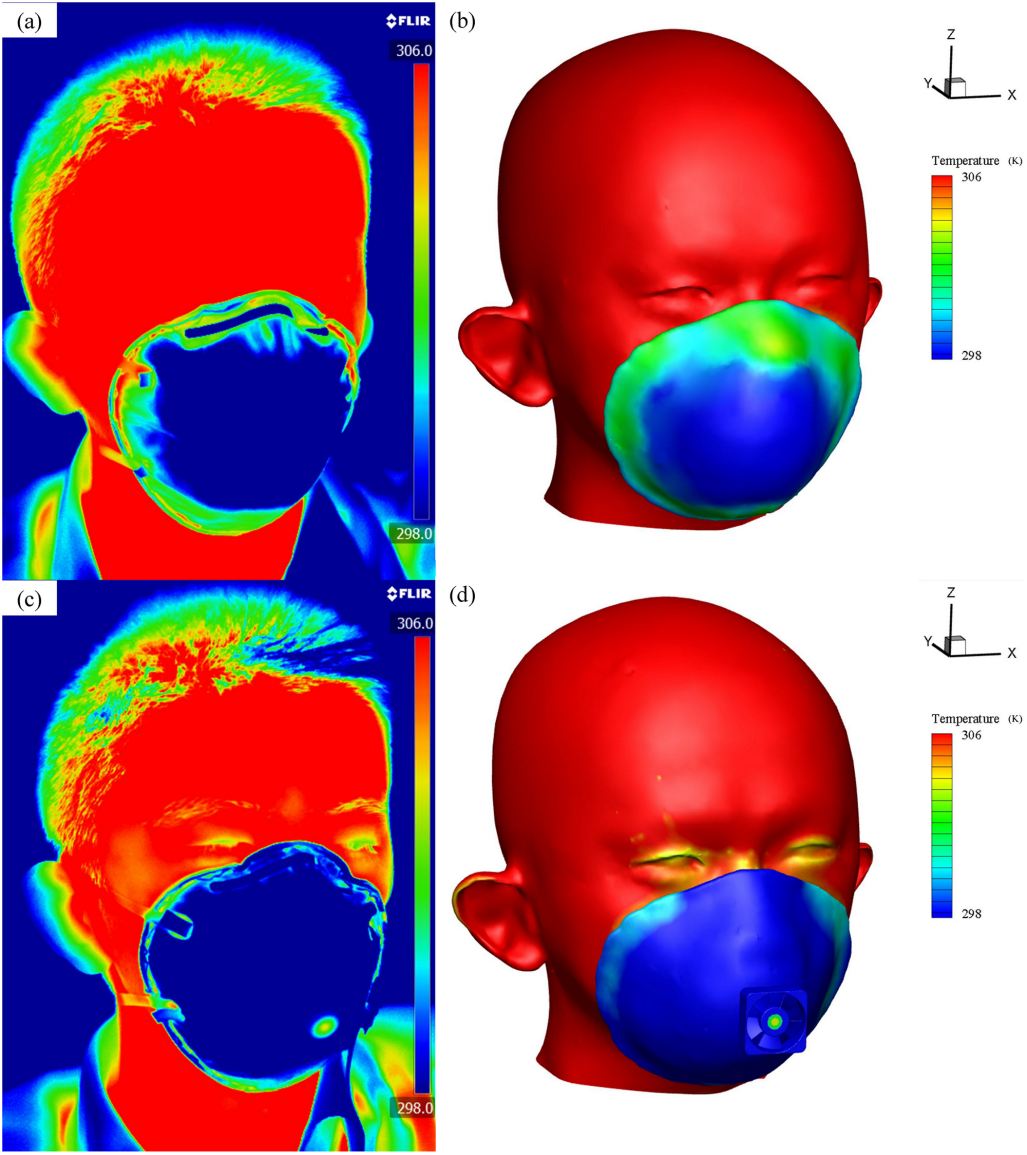
Temperature distribution when FFRs without a ventilation fan (a), (b) and with a ventilation fan (c), (d).

Fig 8 shows the temperature distribution of the headform and FFR measured by the FLIR A655sc infrared camera and CFD simulation. Results are taken at the end of the 20th typical inhalation. The face has a relative higher temperature than the FFR surface, at up to 306 K. The surface temperature of the FFR without a ventilation fan is unevenly distributed both in experimental (Fig 8(a)) and simulation (Fig 8(b)) results. Moreover, the temperature is higher at the edge of the FFR, which is in contact with the warm face. It gradually becomes lower towards the middle of the FFR, and finally close to the environment temperature at 298 K, because the inhalation dissipates the heat and then the fresh air occupies this zone. In Fig 8(c) and 8(d), the FFR with a ventilation fan is worn by the same subject at the same time point. Both experimental and simulated fans are rotating at 7,500 rpm, creating airflows towards the FFR and face. It can be found that both in the experiment and simulation, the temperature on the FFR and face is significantly reduced compared to that in Fig 8(a) and 8(b). On the FFR surface, the temperature distribution is more uniform, and the temperature on its edge is lowered by the fan. In addition, the temperature on the face is partially lowered. Specially, zones around eyes and eyebrows appear in yellow, which represents a lower temperature than other face zones appearing in red. It is because these zones are closer to the fan and more heat is eliminated by the fan airflow.

**Fig 9:**
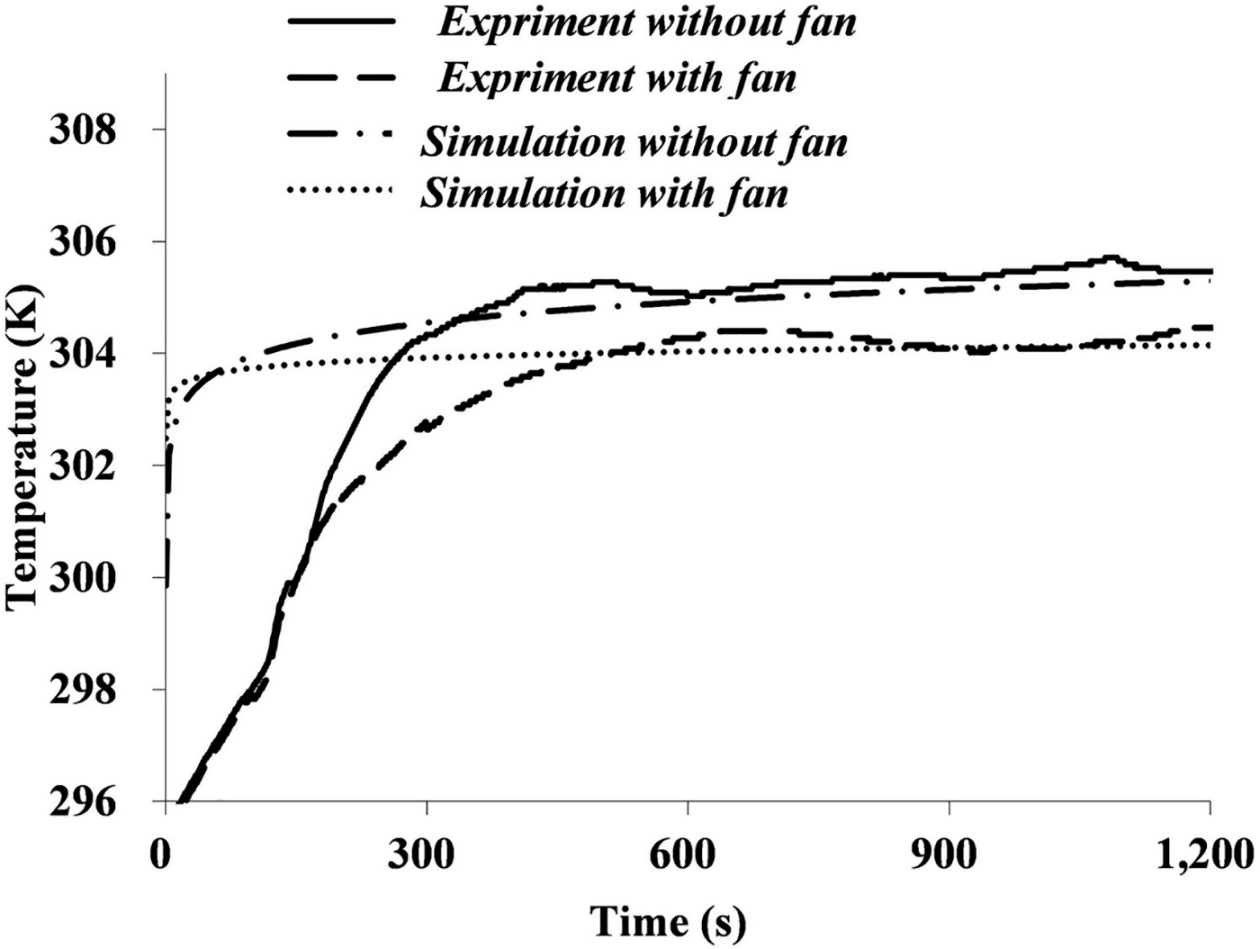
Temperature of FFR deadspace with and without a ventilation fan.

Fig 9 shows a comparison of the deadspace temperature between simulation and experimental results. Each curve follows the same trend that the deadspace temperature accumulates gradually over time and reaches a plateau. The plateaued values from simulation and experimental results show a good consistency. Moreover, results demonstrate that adding an axial fan to the FFR can lower the deadspace temperature by 2 K approximately.

However, there are still some differences between simulation and experimental results. Temperature growth rates of simulation results are larger than those of experimental results. This is probably caused by the simplification of the simulation model; the contribution of the human face and blood to the temperature growth is simplified. Furthermore, plateau values of simulation results are much more stable than those of experimental results. Simulation temperatures show basically no changes once they reach plateau values, whereas experimental temperatures still fluctuate within a limited range. This is probably because that each respiratory cycle simulated by a computer follows same boundary conditions, whereas small variations in the respiratory rate and micro-climate around the subject are inevitable during experimental measurements.

### D) Summary

The CO_2_ level and temperature in a normal FFR deadspace has been proven higher than the ambient level based on this study, our previous study and many other investigations. [5–7, 10–11] Limited research has focused on how to improve this. [10] The most popular method currently available uses an exhalation valve (EV), which has been reported to be effective in lowering the deadspace temperature by 0.1~0.8 K. [2, 22] However, the ventilation provided by the exhalation valve depends on the respiratory rate, and at least 30 L/min airflow is needed to develop streamlined airflows during the exhalation and activate the exhalation valve. [23] In our simulation, 30 L/min is the maximum airflow rate according to Fig 2(c), which is too small to activate the exhalation valve. Indeed, the wearers`respiratory rate could be insufficient to open the EV (at least partially) if they are at sedentary levels or at low work rates, so the FFR with the exhalation valve would not show any difference from the normal FFR in these situations. [24–26]

Therefore, this study presents a novel approach to optimize the flow field of the FFR-respiratory system: an active axial fan is attached for the air ventilation. The essence of this idea is that the driving force should be provided by the FFR itself instead of the exhaled air. So a small battery-driven axial fan is introduced to replace the breathing-driven valve, and it provides greater reliability and flexibility than the exhalation valve. The power is provided by a battery, therefore wearer can switch it on whenever they need, and they do not have to worry that the ventilation efficiency might be lowered by the insufficient exhaled air. In addition, this design leaves the completeness of the filter facepiece unchanged. Therefore, the ventilation fan can be attached to any normal FFR and convert it into a ventilation FFR; the FFR can still be disposable as before and the ventilation fan can be reused to save the cost. This advantage makes the design more feasible and both economically and environmentally robust.

Most importantly, the effectiveness of this design in improving wearers`comfort is demonstrated by the simulation and experiment. The deadspace CO_2_ level is controlled from 3% to 2% according to our simulation results in Fig 7(b). Although a previous study showed better efficiency in CO_2_ controlling and limit the CO_2_ level down to 1%, [10] their design destroyed the FFRs`leakproofness and was less feasible. The possible impact of EVs on lowering deadspace CO_2_ levels remains to be manifest according to a previous research. [1] In addition, this design can reduce the deadspace temperature substantially. To our knowledge, no previous study focuses on using axial fan to lower FFRs`temperature, therefore, EVs were utilized to compare with this design, which were reported to lower the deadspace temperature by 0.1~0.8 K. [2, 22] Both the simulation and experiment suggested that this design can lower the deadspace temperature by 2 K, it is apparently better than the EVs solution.

However, even with all these advantages mentioned before, this design can still be further improved. The axial fan slightly adds some noise during its operation, and the noise value is 17.5 to 25 dB-A according to the official data. Although it is sustainable during our experiment, future work should try to improve this deficiency by ensuring quieter operation.

## Conclusion

This study presents a design of FFR ventilation fan to lower the FFR deadspace CO_2_ level and temperature; CFD simulation and experiment validation was introduced to test this design. With a 3-dimensional model and transient simulation result, the characteristics of this new flow field were determined. The streamline out from the ventilation fan is cup-shaped distributed and fits the shape of the standard N95 FFR well. The simulation also demonstrated that blowing towards the face is a better direction for the ventilation fan according to results of the streamline distribution, deadspace temperature and CO_2_ volume fraction. In agreement with published experimental observations, the standard FFR without a fan gives a rise to the deadspace CO_2_ volume fraction of around 3% in this simulation. In this novel model, the value is lowered to approximately 1% by the ventilation fan. Moreover, based on the infrared image and simulation, the ventilation FFR lowers the FFR surface temperature and face temperature. In addition, based on the simulation and wireless sensor measurement, the deadspace temperature is elevated and reaches a value close to the body temperature when a standard N95 FFR is worn. After the ventilation fan is added, both results from the CFD simulation and temperature sensor suggested that deadspace temperature can be lowered about 2 K.

Therefore, adding an inward-blowing fan on the outer surface of an N95 FFR is a feasible approach to reducing the deadspace CO_2_ concentration and improve temperature comfort.
